# Hepatic transcriptome analyses of juvenile white bass (*Morone chrysops*) when fed diets where fish meal is partially or totally replaced by alternative protein sources

**DOI:** 10.3389/fphys.2023.1308690

**Published:** 2024-01-15

**Authors:** S. Adam Fuller, Jason W. Abernathy, Nithin Muliya Sankappa, Benjamin H. Beck, Steven D. Rawles, Bartholomew W. Green, Kurt A. Rosentrater, Matthew E. McEntire, George Huskey, Carl D. Webster

**Affiliations:** ^1^ USDA-ARS Harry K. Dupree Stuttgart National Aquaculture Research Center (HKDSNARC), Stuttgart, AR, United States; ^2^ USDA-ARS Aquatic Animal Health Research Unit (AAHRU), Auburn, AL, United States; ^3^ Oak Ridge Institute for Science and Education (ORISE), ARS Research Participation Program, Oak Ridge, TN, United States; ^4^ Iowa State University, Agricultural and Biosystems Engineering, Ames, IA, United States

**Keywords:** alternative diets, carnivorous fish, fish meal, plant meal, transcriptomics, RNAseq, white bass, temperate basses

## Abstract

White bass (*Morone chrysops*) are a popular sportfish throughout the southern United States, and one parent of the commercially-successful hybrid striped bass (*M. chrysops* ♂ *x M. saxatilis* ♀). Currently, white bass are cultured using diets formulated for other carnivorous fish, such as largemouth bass (*Micropterus salmoides*) or hybrid striped bass and contain a significant percentage of marine fish meal. Since there are no studies regarding the utilization of alternative proteins in this species, we evaluated the global gene expression of white bass fed diets in which fish meal was partially or totally replaced by various combinations of soybean meal, poultry by-product meal, canola meal, soy protein concentrate, wheat gluten, or a commercial protein blend (Pro-Cision™). Six isonitrogenous (40% protein), isolipidic (11%), and isocaloric (17.1 kJ/g) diets were formulated to meet the known nutrient and energy requirements of largemouth bass and hybrid striped bass using nutrient availability data for most of the dietary ingredients. One of the test diets consisted exclusively of plant protein sources. Juvenile white bass (40.2 g initial weight) were stocked into a flow-through aquaculture system (three tanks/diet; 10 fish/tank) and fed the test diets twice daily to satiation for 60 days. RNA sequencing and bioinformatic analyses revealed significant differentially expressed genes between all test diets when compared to fish meal control. A total of 1,260 differentially expressed genes were identified, with major ontology relating to cell cycle and metabolic processes as well as immune gene functions. This data will be useful as a resource for future refinements to moronid diet formulation, as marine fish meal becomes limiting and plant ingredients are increasingly added as a reliable protein source.

## Introduction

Aquaculture has traditionally used marine fish meal (FM) for the majority of protein in prepared diets due to its excellent nutrient composition, especially for carnivorous fish. However, it is the most expensive macro-ingredient in the diet and is of finite supply. Thus, within the past 4 decades, much research has been conducted to partially or totally replace FM with alternative, more renewable protein ingredients. Both animal-source protein ingredients (such as poultry by-product meal) and plant-source protein ingredients (such as soybean meal and canola meal) have been added to aquaculture diets, partially or totally replacing FM depending upon the fish species. However, with this dietary shift away from FM, there has been evidence that variations in gene expression have been reported. Panserat et al. (2008) reported that a decrease in protein biosynthesis capacity and nitrogen metabolism occurred in rainbow trout (*Oncorhynchus mykiss*) in liver genes, while Panserat et al. (2009) reported that 2% of the hepatic transcriptome were differentially expressed between fish fed a diet containing FM and fish fed a plant-based diet. However, no major dysfunction of hepatic metabolism and no over-expression of hepatic genes involved in stress and health were reported.

Webster et al. (1999) stated that blends of animal and plant protein ingredients in aquafeeds often result in better growth performance in fish because they provide complimentary nutrient profiles, especially vitamins and essential amino acids (EAAs), that meet the animal’s nutrient requirements. Further, they may reduce potential negative palatability (diet acceptability) associated with using predominantly one protein source (Webster et al., 1992). Among plant-protein ingredients, soybean meal (SBM) is the most widely used in aquaculture diets because of its wide availability, good nutrient composition, and high digestibility (Akiyama et al., 1989; Gatlin et al., 2007; Lemos et al., 2009). However, SBM also has negative attributes that may limit its use at high percentages, or as the predominant protein in commercial aquaculture diets for some fish species. For example, SBM has lower overall EAA concentrations than FM (deficiencies in methionine—MET, lysine—LYS, and threonine -THR), and lack the essential n-3 fatty acids eicosapentaenoic acid (EPA; 20:5n-3), and docosahexaenoic acid (DHA: 22:6n-3; Fox et al., 2004; Gatlin et al., 2007; NRC, 2011). Soybean meal also contains anti-nutritional factors (ANFs), such as trypsin inhibitors, lectins, phytic acid, saponins, antivitamins, oligosaccharides, and high levels of non-starch polysaccharides that may affect nutrient digestibility and/or availability to fish (Francis et al., 2001; Gilani et al., 2005; Gatlin et al., 2007).

The second-most widely used plant protein ingredient is rapeseed (canola) meal (CM). Canola is the name for varieties of rapeseed (*Brassica napus* and *B. campestris*) that have low levels of erucic acid and glucosinolates. Canola meal possesses favorable EAA composition, bioactive peptides (Akbari and Wu, 2015), and phenolic compounds that have antioxidant activity (Mazumder et al., 2016). However, use of CM in aquaculture diets is limited by the presence of ANFs and high fiber levels (Higgs et al., 1982; Lim et al., 1998; Webster et al., 2000; Glencross et al., 2004; Ngo et al., 2016; Dossou et al., 2018).

Animal-protein ingredients, such as poultry by-product meal (PBM), have variable success as partial or total alternatives to FM in diets for various fish. For several carnivorous fish, PBM is an excellent replacement for moderate-to-high percentages of FM (Nengas et al., 1999; Tidwell et al., 2005; Wang et al., 2015; Wu et al., 2018). However, animal by-product meals can vary greatly in quality depending upon what is used to make the final product. Further, there are commercial protein blends available for use as FM substitutes in aquaculture diets, such as Aqua-Pak Elite™ series and Pro-Cision™ (H.J. Baker Bros., Inc., Tuscola, TX, United States) blends that are comprised of proprietary combinations of animal proteins that include PBM, meat and bone meal, feather meal, blood meal, and plant proteins that include corn gluten, wheat gluten (WG), distiller’s dried grains with solubles, soy protein concentrate (SPC), and soy protein isolate (SPI) (McCann et al., 2021).

Nutrigenomics, the integration of nutrition and genomic analysis, allows for the understanding of the effects, and affects, of diet on gene expression. This allows for a more thorough and complete assessment of ingredients used in diet formulations for fish. Replacement of FM by other animal and/or plant protein sources is a vital area of research for aquaculture as use of plant-protein ingredients has been shown to affect growth, body composition, and biochemical/histological composition of fish at the molecular level (Gómez-Requieni et al., 2004). Growth rates in fish have been reported to be influenced by growth hormone (GH) and insulin-like growth factor (IGF) axis (Company et al., 2001) and protein sources may affect the expression of GH and IGF-1 encoding genes (Kumar et al., 2011).

White bass (WB; *Morone chrysops*) are an under-utilized fish in the U.S., but has the potential to become a valuable food fish industry. Because it is carnivorous, WB typically are fed high-protein (>35% crude protein) diets formulated with high FM content similar to other commercially important carnivorous fish. Nutrient requirements or diet formulations for WB may be similar to hybrid striped bass (HSB; *M. chrysops* ♂ *x M. saxatilis* ♀ and *M. saxatilis* ♂ *x M. chrysops* ♀) (NRC, 2011) and largemouth bass (LMB; *Micropterus salmoides*) (Tidwell et al., 1996; Coyle et al., 2000; Portz et al., 2001; Tidwell et al., 2005), but to date, there are no published reports of nutrient requirements or diet formulations for WB and thus, no data on gene expression when WB are fed diets in which FM has been replaced. The objectives of this feeding trial were to evaluate global gene expression of white bass fed diets in which FM was partially or totally replaced by a combination of SBM and PBM, and other plant proteins that included CM, SPC, WG, or the commercial protein blend Pro-Cision™.

## Materials and methods

### Fish and culture systems

A total of 180 white bass (40.1 ± 1.83 g; mean ± SE) were randomly stocked into 200-L tanks (*n* = 18) at a density of 10 fish/tank (three replicate tanks per dietary treatment). The experimental protocol was approved by the HKD-SNARC Institutional Animal Care and Use Committee and conformed to USDA Agricultural Research Service Policies and Procedures 130.4 and 635.1. Water from a freshwater well was supplied at 2.0 L/min in a flow-through design. Fish were carefully hand-fed their respective pelleted test diets twice daily (0700 and 1530 h) all they would consume in 20 min for the 60-day feeding trial. No more diet was fed when the last pellet was consumed. Mortalities were replaced during the first week of the feeding trial. To mimic natural environment, light was provided by overhead fluorescent ceiling lights set on a photoperiod of 15 h:9 h light:dark cycle. All tanks were siphoned daily to remove any fecal waste. If present, mortalities were recorded and removed daily. All tanks were covered with polyethylene mesh to prevent fish loss due to escapement or jumping to adjacent tanks.

Dissolved oxygen (DO) and water temperature (°C) were measured daily (Model 556MPS, YSI, Inc., Yellow Springs, OH, United States), while alkalinity, hardness, chlorides, total ammonia nitrogen (TAN), nitrite nitrogen, nitrate, and pH were recorded weekly. TAN and nitrite nitrogen were analyzed by multiparameter colorimeter (Model DR 900, Hach, Co., Loveland, CO, United States) using the salicylate method (TAN) and azo-dye method (nitrite nitrogen). Average water quality parameters (±SE) during the feeding trial were 22.5°C ± 0.1°C water temperature, 7.99 ± 0.03 mg DO/L, 0.03 ± 0.01 mg TAN/L, 0.01 ± 0.00 mg nitrite/L, 191.5 ± 1.9 mg total alkalinity/L, 110.8 ± 1.4 mg hardness/L, 202.1 ± 3.1 mg chloride/L, and a pH of 8.14 ± 0.12. All parameters were within acceptable limits for fish growth and health (Boyd, 1979).

### Diets, feeding, and sampling

Six isonitrogenous (40% protein), isolipidic (11%), and isocaloric (17.1 kJ/g) diets were formulated (Table 1) on an as-fed basis to meet the known nutrient and energy requirements of LMB (Portz and Cyrino, 2003; Portz and Cyrino, 2004). Diets were formulated on a digestible protein basis or an available amino acid basis when available for FM, SBM, PBM, and wheat as determined in LMB (Portz and Cyrino, 2004), WG (Joana et al., 1998) and soy protein concentrate (Kaushik, 2002) as determined in European seabass, and CM as determined in HSB (Gaylord et al., 2004). Amino acid availabilities for WG, SPC, and CM in white bass are not published and were assumed to be 90%. Nutrient requirements for LMB were used as it has been previously demonstrated that LMB can be fed a diet formulated to meet nutrient requirements of HSB (Tidwell et al., 2005) and LMB require some EAA at higher levels than HSB (NRC, 2011): THR (Rahman et al., 2021), LYS (Dairiki et al., 2007), and ARG (Zhou et al., 2012). EAA composition of each formulated diet is provided in Table 2. Lipid contribution from dry ingredients was balanced with canola oil and menhaden fish oil to maintain the diets isolipidic and isoenergetic, while the contribution of fish oil to total lipid was maintained constant in all diets by including 1.5%–2% menhaden fish oil in the fish meal free diets. Diet 1 (30% FM-control) was formulated as a high-quality, commercial carnivorous fish diet containing 30% FM and 37% SBM. Diets 2–5 were formulated to replace FM partially or totally with other commercial protein ingredients. Diet 6 (Pro-Cision™ + SBM) was formulated to replace FM completely with a commercial protein blend (AquaPak Pro-Cision™, H.J. Bakers Bros., Inc., Tuscola, TX, United States) at a 40% inclusion level to have similar formulated nutrient composition as the control (Diet 1).

**TABLE 1:** Composition (g/kg; as-fed basis) of six practical test diets fed to juvenile white bass. Values are means of three determinations per diet.

Ingredient	Diet					
	1 (30%FM-control)	2 (15%FM + SBM)	3 (15%FM + PBM + SBM)	4 (PBM + SBM)	5 (all-plant)	6 (Pro-Cision™ + SBM)
FM ^a^	300.00	150.00	150.00	0.00	0.00	0.00
PBM ^a^	0.00	0.00	160.00	320.00	0.00	0.00
SBM ^a^	370.00	560.00	340.00	310.00	390.00	200.00
CM ^a^	0.00	0.00	0.00	0.00	100.00	0.00
SPC ^a^	0.00	0.00	0.00	0.00	150.00	0.00
WG ^a^	0.00	0.00	0.00	0.00	50.00	0.00
Pro-Cision™ ^a^	0.0	0.00	0.00	0.00	0.00	400.00
Wheat ^a^	220.10	160.60	240.60	270.20	170.90	300.60
Canola oil ^a^	50.00	40.50	20.50	4.00	30.70	0.00
Menhaden fish oil ^a^	30.00	40.50	40.50	60.00	60.00	60.00
Dicalcium phosphate	20.00	20.50	20.50	20.50	20.50	20.50
Choline chloride	3.00	3.00	3.00	3.00	3.00	3.00
Stay C^©^ (35%) ^a^	1.00	1.00	1.00	1.00	1.00	1.00
Vitamin mix ^b^	4.00	4.00	4.00	4.00	4.00	4.00
Mineral mix ^c^	1.00	1.00	1.00	1.00	1.00	1.00
Analyzed composition ^d^						
Moisture (%)	90.94	60.97	60.22	70.11	60.63	70.56
Protein (%)	410.14	400.30	400.27	380.90	400.60	420.90
Lipid (%)	100.86	100.55	100.75	100.18	100.69	90.82
Fiber (%)	10.55	20.75	10.65	10.60	30.35	10.10
Ash (%)	90.38	90.08	100.67	110.97	70.19	90.34
Available P ^e^	6.60	5.60	7.20	7.40	4.40	13.20

^a^ Ingredient designations and sources: FM, Special Select™ menhaden fish meal (Omega Protein Corp., hammond, LA, United States); PBM, petfood grade poultry by-product meal (Tyson Foods, Inc., sedalia, MO, United States); SBM, soybean meal (Dakotaland Feeds, Huron, SD, United States); CM, canola meal, (Rangen, Buhl, ID); SPC, soy protein concentrate (Profine VF, Solae Inc., Minneapolis MN, United States); WG, wheat gluten meal (Rangen, Buhl, ID, United States); Aqua-Pak Pro-Cision™ (HJ, baker brothers, Inc., tuscola, TX, United States); wheat (Rangen, Buhl, ID, United States); canola oil, (Rangen, Buhl, ID); menhaden fish oil (Omega Oils, Reedville, VA, United States); Stay C^©^35(DSM, nutritional products, Basel, Switzerland).

^b^ Vitamin mix supplied the following (mg or IU/kg of diet): biotin, 0.64 mg; B_12_, 0.06 mg; E (as alpha-tocopherol acetate), 363 IU; folacin, 9.5 mg; myo-inositol, 198 mg; K (as menadione sodium bisulfate complex), 3.7 mg; niacin, 280 mg; d-pantothenic acid, 117 mg; B_6_, 31.6 mg; riboflavin, 57.4 mg; thiamin, 35.8 mg; D_1_, 440 IU; A (as vitamin A palmitate), 6607 IU.

^c^ Mineral mix supplied the following (g/kg of diet): zinc, 0.07 g; manganese, 0.02 g; copper, 0.002 g; iodine, 0.010 g.

^d^ Dry-matter basis.

^e^ Estimated from availability of P in dietary ingredients for hybrid striped bass according to Barrows et al. (2016). All diets met P requirement for hybrid striped bass (NRC, 2011).

**TABLE 2:** Essential amino acid (EAA) composition (g/kg; as-fed basis) of six test diets fed to juvenile white bass. Values are means of two determinations per diet.

	Diet					
Amino acid	1 (30%FM-control)	2 (15%FM + SBM)	3 (15%FM + PBM + SBM)	4 (PBM + SBM)	5 (All-plant)	6 (Pro-Cision™ + SBM)
Essential						
ARG	23.6 ± 1.1	23.8 ± 1.5	32.4 ± 1.5	27.7 ± 1.5	23.6 ± 0.2	21.5 ± 1.8
HIS	11.9 ± 0.6	12.3 ± 0.5	14.8 ± 0.7	13.3 ± 0.7	12.2 ± 0.1	12.5 ± 1.2
ILE	15.9 ± 0.6	15.4 ± 0.9	20.6 ± 1.0	16.5 ± 0.9	14.5 ± 0.4	19.1 ± 0.6
LEU	28.8 ± 1.1	27.6 ± 1.4	38.0 ± 1.8	30.8 ± 1.6	27.8 ± 0.1	27.2 ± 1.9
LYS	28.3 ± 1.3	28.2 ± 1.3	24.0 ± 2.0	24.5 ± 1.3	24.1 ± 2.8	22.8 ± 1.5
MET	7.4 ± 1.7	6.7 ± 0.3	6.8 ± 1.0	6.1 ± 0.7	5.6 ± 2.6	8.3 ± 3.3
PHE	19.6 ± 1.1	17.5 ± 0.9	22.5 ± 4.0	18.7 ± 0.8	17.8 ± 0.1	18.0 ± 0.9
THR	18.5 ± 0.8	17.0 ± 0.9	22.8 ± 1.1	18.3 ± 0.9	16.6 ± 0.7	14.8 ± 1.2
VAL	18.4 ± 0.9	18.2 ± 0.8	24.3 ± 1.2	19.0 ± 1.0	16.9 ± 1.3	18.7 ± 1.9

All ingredients were ground in a pilot-scale hammermill (Viking, CPM Roskamp Champion, Waterloo, IA, United States), and then blended in a rotating mixer (Model 043206, Kobalt/Monarch Industries, Winnipeg, Canada). Extrusion was performed using a single-screw extruder (Model 500, Insta-Pro, Inc., Des Moines, Iowa, United States) with a 45 mm diameter screw and a 20:1 screw length to diameter (L/D) ratio. Feed blends were manually fed into the extruder at a constant rate. The extruder was connected to a 7.5 HP motor and screw speed was set at 600 RPM. A circular die plate (with multiple 3 mm holes) was attached to the extruder. The mass flow rate was determined by collecting extrudate samples at 30 s intervals during extrusion processing and weighing the samples on an electronic balance. Observed mass flow rates ranged from 0.089 to 0.095 kg/s. The temperatures of the die and of the resulting pellets were recorded every 2 minutes using an infrared thermometer and determined to be 53°C ± 5°C for the die plate and 63°C–70°C for the extrudates. After extrusion processing, the extrudates were air dried for 24 h, then bagged and frozen. Diets were stored at −20°C in plastic containers until fed.

### Data collection and sample analysis

Fish were not fed for 18-h prior to the conclusion of the study. Subsequently, fish in each tank were enumerated to determine survival (%), individually weighed to the nearest 0.01 g on an electronic scale (Model AB54-S; Mettler Toledo, Columbus, OH, United States), and measured for total length to the nearest 0.1 mm. Three fish from each of the three replicate tanks each diet (*n* = 9) were arbitrarily selected, euthanized according to American Veterinary Medical Association guidelines (Underwood and Anthony, 2020), and liver samples dissected and placed in RNA*later* Stabilization Solution (ThermoFisher Scientific, Waltham, MA) and stored at −80°C for transcriptomic analysis.

### RNA isolation from tissues and creation of sequencing libraries

Total RNA was extracted with the Qiagen RNeasy Mini Kit (Cat. No. 74106; Germantown, MD) per the manufacturer’s instruction. Total RNA was treated with Amplification Grade DNase I (Cat. No. AMPD1; Sigma-Aldrich, St. Louis, MO) per the package insert. Afterword, each sample was ethanol-precipitated and resuspended in nuclease-free water. Sample concentrations were then measured via spectrophotometry (Bio-Tek Synergy H1, Winooski, VT) and each sample standardized to 100 ng/μL by dilution with nuclease-free water. RNA quality was assessed using the Agilent BioAnalyzer with the RNA 6000 Nano Kit (Cat. No. 5067-1511; Santa Clara, CA), where RNA Integrity Numbers (RIN) > 9 were considered high-quality for processing the RNA further to be sequenced. Sequencing libraries were then prepared using the NEBNext Ultra II Directional RNA Library Prep Kit for Illumina (Cat. No. E7760L) with the NEBNext Poly(A) mRNA Magnetic Isolation Module (Cat. No. E7490L; New England Biolabs, Ipswich, MA). Barcodes used were the NEBNext Multiplex Oligos for Illumina (Index Primers Sets 1 and 2; Cat. Nos. E7335L and E7500L, respectively). Library insert size and quality were assessed on the Agilent BioAnalyzer using the High Sensitivity DNA Kit (Cat. No. 5067-4626). The NEBNext Library Quant Kit for Illumina (Cat. No. E7630L) was used to quantify each sample. Equimolar amounts (5 nM) of indexed samples were combined, and these pooled libraries were sent for 150 bp paired-end sequencing on an Illumina HiSeq X Ten (San Diego, CA) via a service provider (Novogene, Sacramento, CA).

### Curation of the protein-coding white bass transcriptome

A total of 185,531 published white bass transcriptome contigs (Li et al., 2014) were re-analyzed and a curated set of protein-coding transcripts selected to detect differentially expressed genes (DEGs), herein referred to as white bass transcriptome. To create the WB transcriptome, all transcripts were analyzed by BLASTx to the non-redundant (*nr*) database and only those with blast score E-value < e-20 were retained. Then, several measures were taken to reduce redundancy. First, contigs with >90% similarity were clustered using CD-HIT-EST (Fu et al., 2012). Sequence similarity was further detected using BLAT (Kent, 2002), where duplicates at a >80% Similarity threshold were removed. Then, custom shell scripts were used to manually curate these clusters, where those with top-hit BLASTx returns of hypothetical/unknown protein identities were removed, and those with duplicate top-hit descriptions were reduced to include only the longest sequence. This produced a protein-coding WB transcriptome containing 20,914 sequences. BUSCO analysis (Simão et al., 2015) against the Actinopterygii_odb9 lineage dataset indicated a substantially completed transcriptome, with only 8.4% reported as missing.

### Identification of differentially expressed genes

Raw RNAseq reads were processed for quality control using the TrimGalore! Software (Krueger et al., 2021). This software was used to trim low-quality ends from reads and for Illumina adapter removal. Bowtie2 software (Langmead and Salzberg, 2012) was used for alignment of reads to the curated WB transcriptome using the alignment parameters as recommended by the eXpress software (Roberts and Pachter, 2013). Alignment files were piped directly into the eXpress software using the default parameters to generate read-counts. Effective read counts output from the eXpress software were used to calculate pairwise fold-change using the DESeq2 package (Love et al., 2014) of R-bioconductor. A transcript that met the criteria of greater than a 1.5-fold-change difference with a *p*-value of *p* < 0.05 after adjusting for multiple comparisons testing was considered a significant differentially expressed gene (DEG).

### Gene ontology and enrichment analysis

For Gene Ontology (GO) enrichment analyses, WB transcriptome sequences were uploaded into the OmicsBox program (OmicsBox, 2019). Transcripts were functionally re-annotated by BLASTx (E-value < 1E-3) searches to the *nr* protein sequence database at the NCBI and mapped to GO terms where available. This list provided a “reference set” for enrichment analyses. Significant DEG lists between each comparison were used as a “test set” for enrichment analysis to the “reference set” by two-sided Fisher’s Exact Test. Enrichment of all the three major GO categories (Biological Process (BP), Molecular Function (MF) and Cellular Component (CC)) was determined. Data was considered statistically significant using a False Discover Rate (FDR) of FDR <5%. Gene Set Enrichment Analysis (GSEA) was also performed as we have previously detailed (Lange et al., 2018; Lange et al., 2019), where significance was set at FDR <5% after 1,000 permutation tests.

### Quantitative PCR validation of RNAseq

Reverse transcription quantitative PCR (RT-qPCR) was used to validate RNAseq analyses. Each RNA was quantified by spectrophotometry (Bio-Tek). High-quality RNA samples (RIN >9) from liver tissues extracted previously were used for reverse transcription for all the experimental diet samples. The NEB LunaScript RT SuperMix Kit (Ipswich, MA) was used for the synthesis of cDNA from RNA samples. Total reaction volume was 20 μL, including 4.0 μL of LunaScript RT SuperMix (5X), 200 ng template RNA, and the volume adjusted to 20 μL with nuclease-free water. Reaction conditions were as follows: 25°C for 2 min, followed by 55°C for 10 min and heat inactivation at 95°C for 1 min. After reverse transcription, cDNA was stored at − 20°C until usage for RT-qPCR.

The expression levels of liver for different diets were evaluated by RT-qPCR using a 384-well Roche LightCycler 480 (Indianapolis, IN). A total of 14 genes were selected to evaluate the DEGs. Primer sets were either designed in-house using the Primer3 software (Untergasser et al., 2012) or were taken from the literature (Cooper et al., 2006; Childress et al., 2016), as indicated (Supplementary Table S1). RT-qPCR was performed in 10 μL reaction in triplicate for each biological replicate. All nine biological replicates were used in the evaluation. Each 10 µL RT-qPCR reaction consisted of 5 μL of the Luna universal qPCR Master Mix (2X), 0.5 μL of each primer (1 μM), 3 μL of nuclease-free water, and 1 μL of cDNA that had been diluted 1:10 with nuclease-free water. Thermal cycling conditions used were as suggested following the Luna NEB protocol: initial denaturation at 95°C for 15 s, followed by 45 cycles of 95°C for 15 s and 60°C for 30 s. Melting curve analyses was performed on all RT-qPCR runs. No RT enzyme controls, and no template controls were included. Two genes (18S rRNA and β-actin) were evaluated for use as housekeeping genes for data normalization. The 18S rRNA gene was selected, as the cycle threshold (Ct) values for all replicates was <1 Ct variation. For relative gene expression analyses, target samples were processed concurrently with the reference gene and the relative expression was given by using the 2^−ΔΔCT^ method (Pfaffl, 2001). Since the standard curve was between 0.9 and 1.0, it was assumed that the accuracy of primer efficiency for computation was 100% for all targets and the reference gene.

## Results

### Growth and diet utilization

The highest final weight, weight gain, SGR, and lowest FCR were observed for fish fed Diet 4 (PBM + SBM). The lowest final weight, weight gain, SGR, and highest FCR were observed for fish fed Diet 6 (Pro-Cision™ + SBM). Although final individual weight, percentage weight gain, and SGR of fish fed Diet 6 was significantly lower (*p* ≤ 0.05) than those of fish fed Diet 4, fish fed the other diets (Diets 1–3; Diet 5) did not significantly differ (*p* > 0.05) from each other (Table 3). Fish fed Diet 5 (all-plant) had significantly lower final weight and SGR compared to fish fed Diet 4 (PBM + SBM), but were not different from fish fed the other test diets. FCR was not significantly different among dietary treatments and averaged 1.74 (Table 3).

**TABLE 3:** Growth and feed conversion of juvenile white bass (initial average weight, 40.1 ± 1.83 g) fed six diets containing various percentages of fish meal. Means (±SE) within a row with different letters are different (*p* < 0.05). Data adapted from Rawles et al. (2022).

Diet						
Ingredient	1 (30%FM- control)	2 (15%FM + SBM)	3 (15%FM + PBM + SBM)	4 (PBM + SBM)	5 (all-plant)	6 (Pro-Cision™ + SBM)
Final weight (g)	86.70^abc^	92.71^ab^	84.71^bc^	101.49^a^	78.69^bc^	74.35^bc^
Weight gain (%)	130.5^ab^	160.4^ab^	142.8^ab^	183.7^a^	142.9^ab^	102.5^b^
SGR^1^ (%/d)	1.34^abc^	1.56^ab^	1.31^abc^	1.60^a^	1.19^bc^	1.1^c^
FCR^2^	1.78^a^	1.62^a^	1.77^a^	1.56^a^	1.71^a^	1.98^a^

^1^ SGR, specific growth rate.

^2^ FCR, feed conversion ratio.

Calculation formulas are reported in Rawles et al. (2022).

### Differential gene expression

**FIGURE 1:**
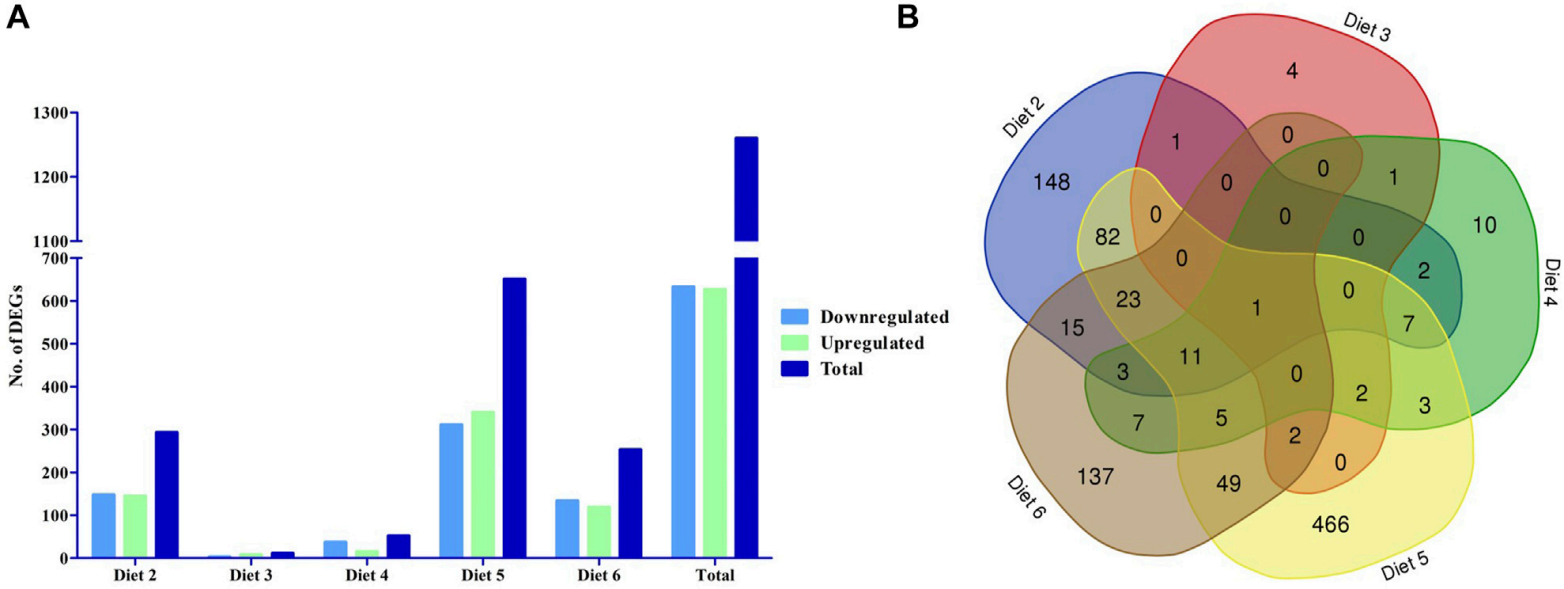
The number of differentially expressed genes (DEGs) in the liver of white bass fed a diet containing soybean meal (SBM-Diet 2), poultry by-product meal (PBM- Diet 3), soy protein concentrate (SPC- Diet 4), canola meal (CM- Diet 5), and commercial protein blend (Pro-Cision™- Diet 6) relative to white bass fed a diet composed predominantly of prime FM (Diet 1). **(A)** The number of differentially expressed genes between experimental diets, the blue filled are down regulated DEGs green filled are upregulated DEGs and the dark blue filled are total DEGs (FDR< 0.05; Fold-change >1.5), and **(B)** Venn diagram showing the number of unique and shared gene cluster in five experimental diets.

We evaluated differential gene expression (DEG) of all dietary treatments, with all treatments being compared to Diet 1 (FM control), with all comparisons as follows: Diet1:Diet 2, Diet1:Diet 3, Diet1:Diet 4, Diet1:Diet 5, Diet1:Diet 6. We found a total of 1,260 DEGs that were differentially expressed greater than 1.5-fold magnitude in our dietary comparisons (Figures 1A, B; Table 4). For the Diet1:Diet 2 comparison, 293 DEGs were identified, including 148 down-regulated and 145 up-regulated genes (Supplementary Table S2). For the Diet1:Diet 3 comparison, 11 DEGs were identified, including 3 down-regulated and eight up-regulated genes (Supplementary Table S3). For the Diet1:Diet 4 comparison, 52 DEGs were identified, including 37 down-regulated and 15 up-regulated genes (Supplementary Table S4). For the Diet1:Diet 5 comparison, 651 DEGs were identified, including 311 down-regulated and 340 up-regulated genes (Supplementary Table S5). For the Diet1:Diet 6 comparison, 253 DEGs were identified, including 134 down-regulated and 119 up-regulated genes (Supplementary Table S6).

**TABLE 4:** Differently expressed genes between different dietary treatment combinations (Diets 1–6). All dietary treatments were compared to Diet 1 - fish meal (FM) control. Values indicate contigs/genes passing cutoff values of fold change ≥1.5 (*p* < 0.05) and read number per contig ≥5. Diet treatments include: Diet: **1** (30%FM-control); **2** ((15%FM + SBM)); **3** (15%FM + PBM + SBM); **4** (PBM + SBM); **5** (All-plant); **6** (Pro-Cision™ + SBM).

	1:2	1:3	1:4	1:5	1:6
Up-regulated	145	8	15	340	119
Down-regulated	148	3	37	311	134
Total	293	11	52	651	253

GO analysis was performed on the genes underlying each of these diet treatment comparisons previously listed. Genes were mapped to GO terms and then two-sided Fisher’s exact test (FET) was used to assess significance between comparison genes and the WB transcriptome. While significant (FDR <0.05) representation of ontology could not be found using FET for the Diet1:Diet2, Diet1:Diet3, and Diet1:Diet4 comparisons, upon further examination with GSEA, several genes from the Diet1:Diet2 comparison mapped to BP (Table 5) GO terms relating to cell cycle; Diet1:Diet4 for BP GO terms relating to metabolic and cell process and MF GO terms relating to catalytic, cofactor, anion, and small molecule binding (Table 5); Diet1:Diet6 for CC GO terms relating to mitochondrial inner membrane, organelle inner membrane and mitochondrion were downregulated along with MF GO terms of proton transmembrane transporter activity and BP GO terms of iron and transition metal ion transport. Cellular component (Table 5). In Diet1:Diet2, 14 genes were upregulated, and these genes were involved in the GO terms relating to cell cycle BP for the progression in vascular smooth muscle cell (Supplementary Table S7). BP and MF GO terms were upregulated in the Diet1:Diet4 for DNA replication (three genes), Catalytic activity (two genes), and organophosphate metabolic process (three genes) as provided in Supplementary Table S7. In Diet1:Diet6, 40 genes were downregulated with 30 genes corresponds to CC, six genes to BP and four genes to MF (Supplementary Table S7). Significant (FDR <0.05) perturbation of immune pathways (Table 5) based on FET was observed in the Diet1:Diet5 comparison; out of 28 genes underlying the significant GO, 18 genes were upregulated and primarily represented chemokines, erythropoietin, MHC II, toll-like receptors, and insulin-like growth factor, while ten genes were downregulated. GO analyses revealed genes underlying the Diet1:Diet6 comparison involved in the glutathione biosynthetic process and non-ribosomal peptide biosynthetic process (Table 5); out of twenty genes, six genes were upregulated and 14 genes were downregulated (Supplementary Table S7). These pathways are involved in cellular metabolism, and more specifically in amino acid biosynthesis and protein translation.

**TABLE 5:** Significant descriptions for GO categories within each of the dietary comparisons found to be significantly different than the reference transcriptome following GO mapping, Gene Set Enrichment Analysis (GSEA), and a two-sided Fisher’s exact test (FET) are listed below. A complete listing of all descriptions is provided in Supplementary Tables S2–S6. Diet: **1** (30%FM-control); **2** ((15%FM + SBM)); **3** (15%FM + PBM + SBM); **4** (PBM + SBM); **5** (All-plant); **6** (Pro-Cision™ + SBM). As per GSEA, Diet1:Diet3 and Diet1:Diet5 were not significant whereas in case of FET, Diet1: Diet2; Diet1:Diet3 and Diet1:Diet4 were not significant.

Statistics	Comparison	GO Category	Representation	GO ID	Description	Number of genes	FDR
Gene Set Enrichment Analysis (GSEA)	Diet1:Diet2	BP	TOP	GO:0007049	Cell cycle	14	0.0104
	Diet1:Diet4	BP	TOP	GO:0006259	DNA metabolic process	3	0.0499
		BP	TOP	GO:0006261	DNA-templated DNA replication	3	0.0280
		BP	TOP	GO:0006260	DNA replication	3	0.0208
		BP	TOP	GO:0019637	Organophosphate metabolic process	3	0.0169
		MF	TOP	GO:0140640	Catalytic activity, acting on a nucleic acid	2	0.0361
	Diet1:Diet6	CC	BOTTOM	GO:0005743	Mitochondrial inner membrane	7	0.0203
		CC	BOTTOM	GO:0019866	Organelle inner membrane	7	0.0126
		CC	BOTTOM	GO:0005739	Mitochondrion cellular component	9	0.0147
		CC	BOTTOM	GO:0031966	Mitochondrial membrane	7	0.0288
		BP	BOTTOM	GO:0000041	Transition metal ion transport	3	0.0361
		MF	BOTTOM	GO:0015078	Proton transmembrane transporter activity	4	0.0304
		BP	BOTTOM	GO:0006826	Iron ion transport	3	0.0281
Fisher’s Exact Test	Diet1:Diet5	BP	OVER	GO:0002376	Immune system process	28	0.0299
	Diet1:Diet6	BP	OVER	GO:0006750	Glutathione biosynthetic process	3	0.0185
		MF	OVER	GO:0009055	Electron transfer activity molecular function	8	0.0223
		BP	OVER	GO:0019184	Non-ribosomal peptide biosynthetic process	3	0.0245
		MF	OVER		Oxidoreduction-driven active transmembrane transporter activity	6	0.0489

**FIGURE 2:**
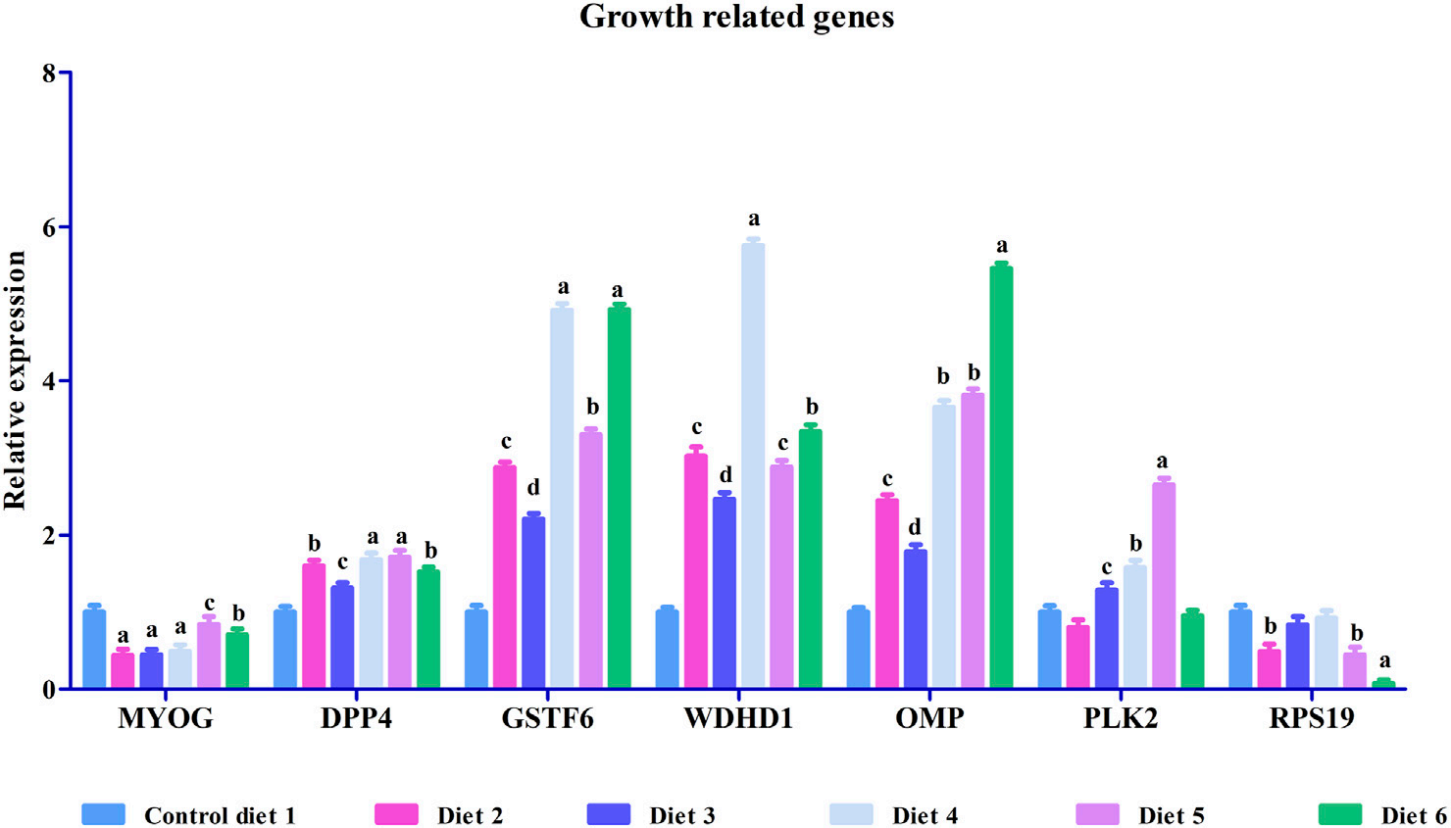
Relative mRNA expression of growth-related genes such as myogenin (MYOG), dipeptidyl peptidase 4-like (DPP4), glutathione S-transferase omega-1 (GSTF6), WD repeat and HMG-box DNA-binding protein1 (WDHD1), olfactory marker protein (OMP), serine/threonine-protein kinase PLK2 isoform 1 (PLK2) and 40S ribosomal protein S19 (RPS19) in liver in white bass fed with different experimental diets as measured by quantitative real-time PCR. Data are presented as mean ± SE (*n* = 9). Different alphabetical letters indicate significant difference with *p* < 0.05.

**FIGURE 3:**
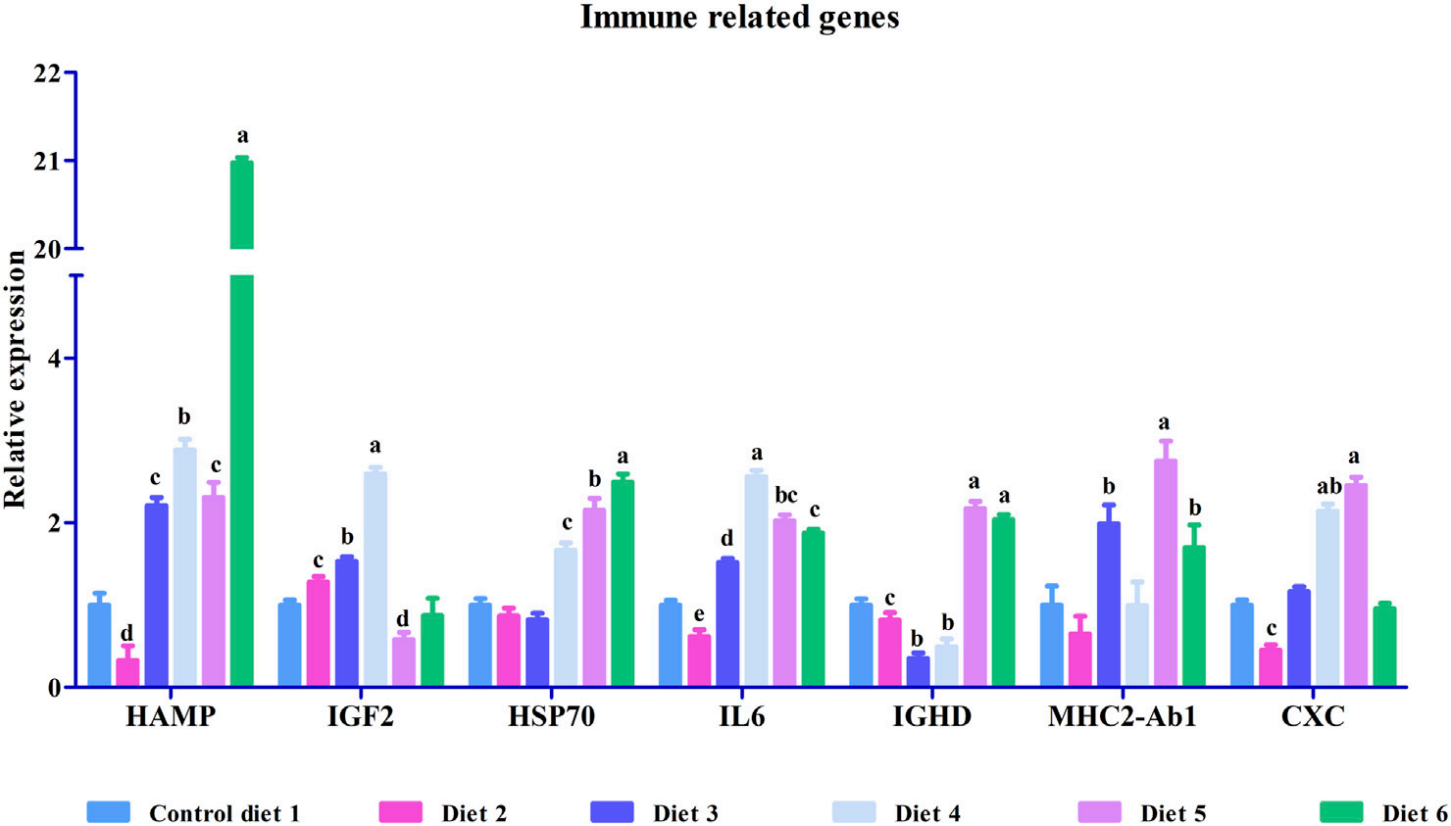
Relative mRNA expression of immune related genes such as hepcidin (HAMP), insulin-like growth factor II (IGF2), heat shock protein 70 (HSP70), interleukin-6 receptor subunit beta-like (IL6), immunoglobulin delta heavy chain (IGHD), MHC class II antigen beta chain (MHC2-Ab1) and C-X-C chemokine receptor type 2-like (CXC) in liver in white bass fed with different experimental diets as measured by quantitative real-time PCR. Data are presented as mean ± SE (*n* = 9). Different alphabetical letters indicate the significant difference with *p* < 0.05.

To validate our differential gene expression analyses using RNA sequencing, seven growth-related genes and seven immune related genes (Figures 2, 3) were analyzed by RT-qPCR for all liver samples. For growth-related genes, myogenin (myog) expression in all fish was upregulated in the Diet 1 (30% FM-control) group relative to all other dietary treatments, with similar results observed for 40S ribosomal protein S19 (RPS19) (Figure 2). Serine/threonine-protein kinase PLK2 isoform 1 (PLK2) was significantly upregulated in fish fed Diet 5 (all-plant) and downregulated in fish fed Diet 2 (15%FM + SBM) and Diet 6 (Pro-Cision™ + SBM). Dipeptidyl peptidase 4-like (DPP4), glutathione S-transferase omega-1 (GSTF6), WD repeat and HMG-box DNA-binding protein1 (WDHD1), and olfactory marker protein (OMP) were significantly upregulated when compared to fish fed Diet 1 (30% FM-control). Immune related genes such as hepcidin (HAMP), heat shock protein 70 (HSP70), interleukin-6 receptor subunit beta-like (IL6), immunoglobulin delta heavy chain (IGHD), MHC class II antigen beta chain (MHC2-Ab1), and C-X-C chemokine receptor type 2-like (CXC) were significantly upregulated in fish fed Diet 5 (all-plant protein) except insulin-like growth factor II (IGF2) (Figure 3). In fish fed Diet 6 (Pro-Cision™ + SBM), IGF2 and CXC were significantly downregulated whereas others were upregulated with a significant upregulation in HAMP. Overall, all RNA sequencing data and RT-qPCR results were correlated (Table 6).

**TABLE 6:** Comparison of RNA-seq and RT-PCR fold change in the liver for the diets (1–6).

		Diet1:Diet2		Diet1:Diet3		Diet1:Diet4		Diet1:Diet5		Diet1:Diet6	
Gene	Feature	RT-PCR	RNAseq	RT-PCR	RNAseq	RT-PCR	RNAseq	RT-PCR	RNAseq	RT-PCR	RNAseq
HAMP	GAZY01116470.1	−3.03	ND	2.21	ND	2.88	ND	2.3	2.71	20.98	9.82
DPP4	GAZY01160786.1	1.60	ND	1.31	3.25	1.68	ND	1.71	2.55	1.52	3.58
GSTF6	GAZY01132657.1	2.88	1.56	2.21	1.61	4.91	1.71	3.3	1.59	4.93	2.35
WDHD1	GAZY01165759.1	3.02	ND	2.46	2.33	5.75	ND	2.88	1.67	3.34	ND
OMP	GAZY01122857.1	2.45	2.62	1.78	ND	3.66	3.57	3.82	4.42	5.46	8.71
RPS19	GAZY01160786.1	−2.04	-7.81	−1.19	ND	−1.09	−8.49	−2.27	−21.61	−12.5	−13.20
IGF2	GAZY01148153.1	1.28	ND	1.53	ND	2.59	ND	−1.72	−1.84	−1.15	−1.56
HSP70	GAZY01110915.1	−1.15	ND	−1.22	ND	1.66	ND	2.15	1.75	2.49	ND
PLK2	GAZY01152818.1	−1.25	−1.89	1.29	ND	1.58	ND	2.65	1.68	−1.05	−1.69
IL6	GAZY01162268.1	−1.61	ND	1.52	ND	2.56	ND	2.02	1.80	1.87	ND
IGHD	GAZY01140595.1	−1.22	ND	−2.86	ND	−2.04	ND	2.17	2.08	2.04	ND
MHC2-Ab1	GAZY01127610.1	−1.54	ND	1.99	ND	−1.01	ND	2.75	2.43	1.7	ND
CXC	GAZY01140604.1	−2.22	1.90	1.16	ND	2.14	ND	2.45	3.49	−1.04	ND

^a^ Growth related genes such as dipeptidyl peptidase 4-like (DPP4), glutathione S-transferase omega-1 (GSTF6), WD, repeat & HMG-box DNA-binding protein1 (WDHD1), olfactory marker protein (OMP), serine/threonine-protein kinase PLK2 isoform 1 (PLK2) and 40S ribosomal protein S19 (RPS19).

^b^ Immune related genes such as hepcidin (HAMP), insulin-like growth factor II (IGF2), heat shock protein 70 (HSP70), interleukin-6, receptor subunit beta-like (IL6), immunoglobulin delta heavy chain (IGHD), MHC, class II, antigen beta chain (MHC2-Ab1) and C-X-C chemokine receptor type 2-like (CXC).

^c^ ND (Not Determined).

## Discussion

RNA sequencing offers insights into an organism’s gene expression for a specific time and can be a useful analytical tool for identifying gene pathways affected by dietary changes in aquaculture species (Chávez-Calvillo et al., 2010; Shiel et al., 2017; Schroeter et al., 2018; Meiler and Kumar, 2021; Lorenz et al., 2022). This is the first published study to evaluate partial to total replacement of FM in diets for WB and their corresponding effects on gene expression. Rawles et al. (2022) reported that growth performance of WB fed an all-plant diet and a diet containing a commercially-blended protein product were reduced compared to fish fed diets containing FM (control) and a FM-free diet containing SBM and poultry by-product meal (PBM). The aim of this study was to utilize RNA sequencing to evaluate possible differences in gene expression in WB to changes in diet. Mechanisms and diet-related gene pathways that may be responsible for any observed changes are discussed.

### Differentially expressed genes compared to diet 1 (30%FM-control)

Changes in the liver transcriptome with replacement of FM by plant protein sources have been reported in previous studies on several fish species (Geay et al., 2011; Tacchi et al., 2012; Juárez et al., 2021). This is the first published report in which differential liver gene expression of WB fed diets containing different protein sources was analyzed. The study was focused on the liver because it is the main organ involved in the use of nutrients and is the main center of intermediary metabolism in animals (Panserat et al., 2009). In the present study, there were more differentially expressed genes (DEGs) in comparisons to Diet 1 (30%FM-control) when the test diet contained no FM and/or high levels of SBM (Diets 2, 5, and 6; 293, 651, 253 DEGs, respectively). The exception to this is Diet 4 (PBM + SBM), which replaced FM with PBM, where there were only 52 DEGs (Table 4).

To further analyze each of the dietary comparisons, we looked for DEGs potentially involved in metabolic pathways and oxidative stress, immune function, and apoptosis. While there were certainly many more functions impacted, given the nature of the study and the composition of test diets, it seemed most prudent to focus on these areas. For a more detailed look at all DEGs by test diet comparisons, refer to the Supplementary Tables S2–S6.

### Metabolism

The capability of the liver to maintain the balance between the intake and release of glucose plays an essential role in the maintenance of the homeostasis of glucose in the blood (Wu et al., 2001). In this study, glucose and fatty acid metabolism DEGs were mainly affected by the inclusion level of SBM and/or the absence of animal proteins. Specific DEGs in each of these test comparisons using the control diet (Diet 1) as baseline will be discussed in more detail in the following sections.

#### Diet 1 (30%FM-control) vs. Diet 2 (15%FM + SBM)

In this test comparison, several DEGs involved in metabolism were identified in fish fed Diet 2 relative to fish fed Diet 1. The most striking result is the decrease in insulin (INS; -57.33-fold) in fish fed Diet 2 relative to fish fed Diet 1, with this being the largest DEG by magnitude in this comparison. This could demonstrate higher circulating levels of glucose in fish fed Diet 2, necessitating the higher insulin demand. Diet 2 contained the highest amount of SBM inclusion (Table 1) of any of the test diets, and clearly replacement of FM with SBM decreased plasma insulin, as has been observed in other fish species (Zhang et al., 2019). Additionally, 6-phosphofructo-2-kinase/fructose-2,6-bisphosphatase 4 (PFKFB4; −3.12) can reduce hepatic glucose production by increasing glycolysis and inhibiting gluconeogenesis, thereby lowering blood glucose, indicating possible lower glucose demand in fish fed Diet 2 (Wu et al., 2001). Interestingly, this gene is also associated with the adaptation of carnivorous fish to diets including plant meals (Metón et al., 2000). Glucose-6-phosphate 1-dehydrogenase (G6PD; 1.68-fold) is the rate-limiting enzyme of the oxidative pentose phosphate pathway (PPP) (Ge et al., 2020). G6PD gene expression, which plays a role in glucose oxidation and increased gluconeogenesis, has been previously shown to be increased in fish after high-carbohydrate feeding (Jiang et al., 2019). Overall, fish fed Diet 2 had significant differential expression of genes involved in metabolic activity, particularly glucose metabolism (Supplementary Table S2).

#### Diet 1 (30%FM-control) vs. Diet 3 (15%FM + PBM + SBM)

In this test comparison, only 11 genes were differentially expressed in fish fed Diet 3 relative to fish fed Diet 1. Only 2 DEGs are associated with metabolism, including dipeptidyl peptidase 4 (DPP4; 3.25-fold) and trehalase (TREH; 29.33-fold). DPP4 is a well-characterized gene which regulates peptide hormones and regulates the endocrine pathway, and when inhibited enhances postprandial metabolism and can be used to treat hyperglycemia (Trzaskalski et al., 2020).

#### Diet 1 (30%FM-control) vs. Diet 4 (PBM + SBM)

In this test comparison, only 52 genes were differentially expressed in the FM diet relative to FM-free diet, including insulin receptor substrate 2 (IRS2; −1.56-fold) and glucose-6-phosphate 1-dehydrogenase (G6PD; 1.80-fold). These genes are involved in insulin regulation and glucose metabolism. IRS2 has been shown to control adipose tissue function and glucose metabolism as well as mediating insulin’s actions (Turaihi et al., 2018). With IRS2 being downregulated in fish fed Diet 4 relative to fish fed Diet 1, this may indicate that there could be adipose tissue activity in Diet 4-fed fish. As stated earlier, G6PD plays a role in glucose oxidation and gluconeogenesis (Metón et al., 2000), which given the upregulation in fish fed Diet 4 could indicate higher metabolic activity. There was also a higher expression of olfactory marker protein (O2; 3.57-fold) in this study which indicates the acceptability of the SPC, CM and commercial protein blend. Previous studies related to plant diet indicated the higher expression of olfactory proteins raises the potential that various extrinsic variables involving olfactory sensing such as locating food, hunger pains, and even the need to avoid predators, play a substantial part in the control of eating behavior in rainbow trout (Heraud et al., 2022).

#### Diet 1 (30%FM-control) vs. Diet 5 (all-plant)

In this test comparison, several DEGs involved in metabolism were identified in fish fed Diet 5 relative to fish fed Diet 1, including acyl-CoA synthetase family member 2 (ACSS2; -2.77-fold), insulin-like growth factor II (IGFII; −1.84-fold), ATP synthase F(0) complex subunit C3 (ATP5G3; −1.59-fold), acyl-CoA desaturase (FADS2; 6.98-fold), ATP-citrate synthase (ACLY; 5.70-fold), insulin-like growth factor-binding protein 3 (IGFBP3; 2.98-fold), and glucose-6-phosphate 1-dehydrogenase (G6PD; 2.34-fold). ACSS2 plays a key role in lipogenesis (Xu et al., 2018). IGFII plays a key role in cell regulation and growth, and interestingly, while there was a clear difference in expression in the current study between fish fed Diet 1 and fish fed Diet 5 (all-plant), previous studies in tilapia have demonstrated that IGFII was not influenced by diet (Herath et al., 2018). It should be noted, however, that Herath et al. (2018) formulated their FM-free diet to contain PBM; thus, it was not an all-plant diet as in the present study. FADS2 is the key enzyme in long-chain polyunsaturated fatty acid (LC-PUFA) biosynthesis. Importantly to fish fatty acid metabolism, ACLY is involved in the production of acetyl-CoA, before FADS2 converts dietary PUFAs, such as linoleic acid and alpha-linolenic acid into HUFAs, such as DHA.

#### Diet 1 (30%FM-control) vs. Diet 6 (Pro-Cision™ + SBM)

In this test comparison, several DEGs involved in metabolism were identified in fish fed Diet 6 relative to fish fed Diet 1, including fructose-1,6-bisphosphatase 1 (FBP1; -2.34-fold), several cytochrome c oxidases (CCO subunit 1; -1.79-fold; subunit 4, -1.75-; subunit 2, -1.63-fold), several mitochondrial NADH dehydrogenases (subunit 2; -1.82-fold; subunit 5; -1,79-fold), ATP synthase F(0) complex subunit C2 (ATP5MC2; -1.56-fold), glucose-6-phosphate 1-dehydrogenase (G6PD; 2.18-fold), and electron transfer flavoprotein-ubiquinone oxidoreductase (ETFQO; 1.7-fold). CCOs, NADH dehydrogenases, and ATP synthases are involved in the respiratory electron transport chain, while G6PD is involved in the PPP and FBP1 is a key protein in gluconeogenesis via glycolysis. Fish fed Diet 6 had much lower metabolic activity relative to fish fed Diet 1, but exhibited higher glycolytic activity, while the latter fish had higher PPP and ETFQO activity. ETFQO is a key component of the electron transport chain, and it acts as a key linkage between the oxidation of fatty acids and some amino acids to the mitochondrial respiratory chain (Zhang et al., 2006).

### Oxidative stress, immune function, and apoptosis

Multiple links have been made between diet, growth performance, and immune response capability (Burrells et al., 2001; Hossain et al., 2016; Kader et al., 2018). It is in this context that we take a closer look at DEGs within each of these test diet comparisons to the control diet (Diet 1).

One of the initial lines of defense used by fish against infections consisted of small peptides known as antimicrobial peptides (AMPs). Fish hepcidins have been discovered in at least 37 fish species since they were initially discovered and isolated for the first time from the hybrid striped bass (Hirono et al., 2005). The liver and kidneys have high levels of fish hepcidin expression, while the heart, brain, skin, gut, blood cells, spleen, stomach, and muscle have low levels of expression. These hepcidin are short, cysteine-rich peptide discovered in fish in two forms, HAMP1 and HAMP2 (Neves et al., 2017). Hepcidin was found to be upregulated in almost all the diet groups including poultry by-product meal (PBM), soy protein concentrate (SPC), canola meal (CM) and a commercial protein blend (Pro-Cision™), except diet 2 soybean meal (SBM) (Table 6). Previous study has shown that these bass hepcidin can significantly reduce bacteria, fungi and yeast (Lauth et al., 2005; Shen et al., 2019). Further, numerous physiological and pathological processes have been linked to ribosomal proteins, and many of them act as antimicrobial proteins in the body’s response to infections. Ribosomal proteins can function as antimicrobial proteins in eukaryotic innate immune systems. The 40S19 was shown to be significantly downregulated in all the test diets.

#### Diet 1 (30%FM-control) vs. Diet 2 (15%FM + SBM)

In this test comparison, several DEGs involved in immune function and apoptosis were identified in fish fed Diet 2 relative to fish fed Diet 1, with a few of the key DEGs discussed below. The complement regulatory protein DAF blocks complement deposition on host cells, which also acts as a regulator of phagocytosis of cells (Dernstedt et al., 2021). Heat shock protein 70 (HSP70; -2.20-fold) and hypoxia up-regulated protein 1 (HYOU1; -1.84-fold) are key regulators of cellular stress. HSP70 has been well studied in many animal models and plays a key role in protein folding and has an important role in the response to oxidative stress (Turner et al., 1999), and its overexpression usually indicates that cells are suffering from oxidative stress. HYOU1 can induce an unfolded protein response in the endoplasmic reticulum as a response to cellular stress (Rao et al., 2021). Apoptosis-inducing factor 3 (AIFM3; 1.88-fold) is part of a cascade that contributes to mitochondrial cell death and participates in the assembly of the respiratory chain as part of normal mitochondrial bioenergetics (Bano and Prehn, 2018). However, long term exposure to oxidative stress has also been shown to induce apoptosis (Dastor and Dreyer, 2001).

#### Diet 1 (30%FM-control) vs. Diet 4 (PBM + SBM)

In this test comparison, several DEGs involved in immune function and apoptosis were identified in fish fed Diet 4 relative to fish fed Diet 1, a few of which will be discussed further below. Spondin-2 (SPON2; -11.13-fold) expression identifies pathogens and triggers an immune response (Klar et al., 1992). Von Willebrand factor A domain-containing protein 7 (VWA7; -9.84-fold) is part of the major histocompatibility complex class III region, which has traditionally been associated with the inflammation response region of the MHC (Deakin et al., 2006). MPV17-like (MPV17L; -5.94-fold) is located in the membrane of the peroxisome and is involved in the regulation of several reactive oxygen species, including glutathione peroxidase and several catalase genes (Iida et al., 2006). Beta-galactoside-binding lectin-like (BGBP; -3.43-fold) is a negative regulator of the cell cycle and plays a role in T cell immune response (Allione et al., 1998). Each of these immune and cellular stress genes is more upregulated in fish fed Diet 4 than in fish fed Diet 1, indicating that these fish were experiencing cellular stress, possibly due to the presence of dietary factors such as ANFs. Immune response to ANFs has been demonstrated in numerous studies involving alternative formulations, including soy and alternative animal protein diets. Caballero-Solaros et al. (2018) demonstrated changes in numerous immune response genes in Atlantic salmon fed FM, animal byproduct meal, or soy protein concentrate, with more genes differentially expressed in fish fed the plant protein diet. Likewise, Juárez et al. (2021) found higher activation of immune response genes in juvenile Pacific yellowtail (*Seriola lalandi*) fed a 25% SBM feed compared to a FM feed.

#### Diet 1 (30%FM-control) vs. Diet 5 (all-plant)

In this test comparison, several DEGs involved in immune function and apoptosis were identified in fish fed Diet 5 relative to fish fed Diet 1, a few of which will be discussed further below. Tumor necrosis factor ligand superfamily member 12 (TNFSF12; -2.35-fold) works with other proapoptotic TNFSF ligands to facilitate cytotoxicity in many cell types, including activated monocytes, dendritic cells, NK cells, and T cells (Wang et al., 2013). Death-associated protein 1 (DAP1; -1.71-fold) is known to function both in apoptosis and autophagy (Xia et al., 2017). Oxidation resistance protein 1 (OXR1; -1.61-fold) is a gene which has previously been shown to be induced under oxidative stress and is vital for defense against reactive oxygen species (Oliver et al., 2011). Complement C4 (C4; 1.59-fold) is one of the chief constituents of innate immunity which participates in a pathway that is responsible for the immediate recognition and elimination of invading microbes and plays an essential role in both the classical and lectin complement pathways (Wang and Liu, 2021). Macrophage-expressed gene 1 protein (MPEG1; 1.68-fold) is responsible for encoding a protein responsible for host defense against invading pathogens (Benard et al., 2015). Caspase recruitment domain-containing protein 19 (CARD19; 1.69-fold) appears to be involved in the regulation of mitochondrial function which affects pro-inflammatory innate immune responses (Rios et al., 2018). Toll-like receptor 8 (TLR8; 1.82-fold) is one of many toll-like receptors that function by sensing distinct pathogen associated molecular patterns and initiate inflammatory reactions important for innate and adaptive immune responses (Moen et al., 2019). The major histocompatibility complex (MHC) class II molecules (MHC class II; 2.43- fold) involved in the presenting of the processed antigens to the CD4(+) T-lymphocytes for the commencement of the antigen-specific immune response (Deakin et al., 2006) (Table 5 and Supplementary Table S7).

In the current study, there was significantly higher expression of immune genes with the GO analyzed by GSEA. The increased expression of immune related genes in the present study indicates the activation of innate and adaptive immune system, as indicated by significant mRNA expression of genes related to inflammatory responses, including proinflammatory cytokines of TNF-α, IL1β, IL6, and IL8 as these act as biomarkers for the activation of inflammatory responses (Han et al., 2022). Further, the two highest upregulated genes in WB fed all plant proteins were claudin-15 (∼26-fold) and matrix metalloprotease 16 (∼45-fold), which are responsible for tight junction permeability and glucose metabolism, as well as tissue remodeling and immunity. Over our results indicate that an all-plant protein diet greatly perturbs the white bass immune system and inflammasome.

#### Diet 1 (30%FM-control) vs. Diet 6 (Pro-Cision™ + SBM)

In this test comparison, several DEGs involved in immune function and apoptosis were identified in fish fed Diet 6 relative to fish fed Diet 1, some of which will be discussed below. As mentioned earlier, Complement C4 (C4; -1.72-fold) is one of the chief constituents of the complement immune response and apoptosis-inducing factor 3 (AIF3; 1.86-fold) is part of a cascade that contributes to mitochondrial cell death. Complement C5 (C5; -1.65-fold) plays an important role in the inflammatory and cell killing processes of the complement system. Complement C1 is a large complex within the complement that triggers the destruction of invading pathogens via lysis or by stimulation of innate and adaptive immune processes, with C1r-A subcomponent (C1r; -1.73-fold) being the auto-activation subunit following pathogen binding (Almitairi et al., 2018). All of these genes were upregulated in fish fed Diet 6 except AIF3. As mentioned previously, numerous other studies demonstrating fish response to ANFs have been published, including those mentioned earlier in the Diet1:Diet5 comparison.

## Conclusion

Transcriptome analysis of the liver of WB revealed differentially expressed genes with unique gene ontology when fish were fed various alternative diets as compared to a standard, high FM diet. Juvenile WB can be fed diets without fish meal without negative effects on growth, survival, and body composition. However, WB fed an all-plant diet (Diet 5) or a protein blended diet (Diet 6) to replace FM had reduced growth, and diet greatly affected the immune system of fish fed these diets. Partial replacement diets and a diet formulated similar to a commercial diet showed an increased biological process involving metabolic and cellular process related to nutrient utilization. Olfactory genes may be responsive to diet intake and in turn growth differences observed, of which further research on palatability for white bass for boosting feed intake is warranted. These factors, and others reported in the present study, indicate possible reasons for the reduced growth observed in fish fed an all-plant diet and a diet with a commercial protein blended product.

## Data Availability

The datasets generated for this study can be found in the NCBI Gene Expression Omnibus (GEO) repository and can be accessed under the accession number GSE220235. All other data that support the findings of this study have been included in the manuscript and Supplementary Material.
