# Supplementary material for: Hepatic transcriptome analyses of juvenile white bass (Morone chrysops) when fed diets where fish meal is partially or totally replaced by alternative protein sources
Source: Front Physiol. 2024 Jan 15;14:1308690. doi: 10.3389/fphys.2023.1308690 (PMC10822904; doi:10.3389/fphys.2023.1308690)
Supplement: Supplementary file 3 [file Table1.DOCX]

**Supplementary Table 1 Primers used for validation of the transcriptome analysis for the White Bass diets**

| Sl. No. | Description (Abbreviation) | Gene ID | Forward Primer (3’-5’) | Reverse Primer | Product size (bp) | Reference |
| --- | --- | --- | --- | --- | --- | --- |
| 1 | Hepcidin (HAMP) | GAZY01116470.1 | GGCCTGACACCCATGAGAAA | CAGAGCCCTCCAGAATGCAA | 150 | This study |
| 2 | Dipeptidyl peptidase 4-like (DPP4) | GAZY01160786.1 | ATACTGAGGCAGGGAGGAGA | CCAGGATTGTCCACCACTCA | 190 |  |
| 3 | Glutathione S-transferase omega-1 (GSTF6) | GAZY01132657.1 | ACCAAGTTCTTTGGTGGCGA | AGGCCTTGTAGGTGTCCTCA | 190 |  |
| 4 | WD repeat & HMG-box DNA-binding protein1 (WDHD1) | GAZY01165759.1 | ATGCCTTGTGAGAGGAAGCC | CTTTTCCCCGACCGTGATGA | 168 |  |
| 5 | Olfactory marker protein (omp) | GAZY01122857.1 | ATTTCCCAACACTGGACCCC | GCCATTTGAGCGGTGAATCC | 115 |  |
| 6 | 40S ribosomal protein S19 (RPS19) | GAZY01160786.1 | AGGCCACTGCTGTCTTGAAA | CCCATATCACTCCAGTGCCC | 165 |  |
| 7 | Insulin-like growth factor II (IGF2) | GAZY01148153.1 | GGTGAGTTAGTCGCCTGGTC | CAGGTGTGGCAAAGTGTGTG | 180 |  |
| 8 | Heat shock protein 70 (HSP70) | GAZY01110915.1 | GACGAGAACCTGAAGGGCAA | ACAGCTACCAGTGGGCATTC | 198 |  |
| 9 | Serine/threonine- protein kinase PLK2 isoform 1 (PLK2) | GAZY01152818.1 | AGAGTATGCCTAAAGCGGGC | GCCAAAGCCGTACTTGTTGG | 101 |  |
| 10 | Interleukin-6 receptor subunit beta-like (IL6) | GAZY01162268.1 | TCTGATTTGGGAGCCCGTTC | CACAGCTTGTACCGACACCT | 165 |  |
| 11 | Immunoglobulin delta heavy chain (ighd) | GAZY01140595.1 | GCTCTGGATGGGGCTTCAAT | TGACTGGTTACTGCCACACG | 115 |  |
| 12 | MHC class II antigen beta chain (MHC2-Ab1) | GAZY01127610.1 | GCAGACCAACATGGAGGGAT | GAGTGAAGAACGCTGAACGC | 191 |  |
| 13 | C-X-C chemokine receptor type 2-like (CXC) | GAZY01140604.1 | CTCACATCCGTGGAGGGTTC | CAGACTCTGGGTGGCAAACA | 194 |  |
| 14 | Myogenin (myog) |  | GGCCACAATGAGAGAGAAGAG | CCTCTGGTTTGGGTTCATCA | 176 | (Childress et al., 2016) |
| 15 | Βeta-actin (β-actin) |  | CCAGATCATGTTCGAGACCTTC | CACCGGAGTCCATGACAATAC | 148 |  |
| 16 | 18S (18S) |  | TCGCTAGTTGGCATCGTTTATG | CGGAGGTTCGAAGACGATCA | 150 | (Cooper et al., 2006) |
